# *Staphylococcus aureus* and Methicillin-Resistant Coagulase-Negative Staphylococci in Nostrils and Buccal Mucosa of Healthy Camels Used for Recreational Purposes

**DOI:** 10.3390/ani12101255

**Published:** 2022-05-13

**Authors:** Vanessa Silva, Manuela Caniça, Vera Manageiro, Newton Verbisck, María Teresa Tejedor-Junco, Margarita González-Martin, Juan Alberto Corbera, Patrícia Poeta, Gilberto Igrejas

**Affiliations:** 1 Microbiology and Antibiotic Resistance Team (MicroART), Department of Veterinary Sciences, University of Trás-os-Montes and Alto Douro (UTAD), 5000-801 Vila Real, Portugal; vanessasilva@utad.pt; 2 Department of Genetics and Biotechnology, University of Trás-os-Montes and Alto Douro, 5000-801 Vila Real, Portugal; gigrejas@utad.pt; 3 Functional Genomics and Proteomics Unit, University of Trás-os-Montes and Alto Douro (UTAD), 5000-801 Vila Real, Portugal; 4 Associated Laboratory for Green Chemistry (LAQV-REQUIMTE), University NOVA of Lisboa, 1099-085 Lisboa, Portugal; 5 National Reference Laboratory of Antibiotic Resistances and Healthcare Associated Infections (NRL-AMR/HAI), Department of Infectious Diseases, National Institute of Health Dr Ricardo Jorge, Av. Padre Cruz, 1649-016 Lisbon, Portugal; manuela.canica@insa.min-saude.pt (M.C.); vera.manageiro@insa.min-saude.pt (V.M.); 6 Centre for the Studies of Animal Science, Institute of Agrarian and Agri-Food Sciences and Technologies, Oporto University, 4051-401 Oporto, Portugal; 7 Embrapa Beef Cattle, Campo Grande 79106-550, Brazil; newton.verbisck@embrapa.br; 8 Research Institute of Biomedical and Health Sciences, University of Las Palmas de Gran Canaria, 35001 Las Palmas de Gran Canaria, Spain; mariateresa.tejedor@ulpgc.es (M.T.T.-J.); margaritarosa.gonzalez@ulpgc.es (M.G.-M.); 9 CECAV—Veterinary and Animal Research Centre, University of Trás-os-Montes and Alto Douro (UTAD), 5000-801 Vila Real, Portugal; 10 Associate Laboratory for Animal and Veterinary Science (AL4AnimalS), University of Trás-os-Montes and Alto Douro (UTAD), 5000-801 Vila Real, Portugal

**Keywords:** *Staphylococcus aureus*, coagulase-negative staphylococci, methicillin-resistant, camels, antimicrobial resistance

## Abstract

**Simple Summary:**

Animal-associated staphylococci have been isolated in human infections. Therefore, these strains may pose a zoonotic risk in addition to constituting a reservoir for antimicrobial resistance genes. In this study, we isolated *Staphylococcus aureus* and other species of staphylococci from camels used for recreational activities in the Canary Islands. Most *S. aureus* lacked the antimicrobial resistance genes, but some staphylococci species carried the *mec*A gene which confers resistance to methicillin. The carriage of this gene conferring resistance to methicillin in staphylococci isolated from camels may be a public health concern since there is a risk of bacterial transmission to humans during recreational activities. Furthermore, since the Canary Islands are the only camel exporter to the European Union, camels could constitute a source of zoonotic agents to the rest of the European countries.

**Abstract:**

Several different species of animals host staphylococci as normal microbiota. These animals can be a source of staphylococci zoonotic infections. People with routine or occupational exposure to infected/colonized animals are at risk of a potential transmission. Therefore, we aimed to investigate the presence of *S. aureus* and other staphylococci in camels used for recreational purposes as well as their antimicrobial resistance, virulence factors and genetic lineages. A total of 172 samples were collected from 86 healthy camels (nose and mouth) from different farms located in the Canary Islands, Spain. Antimicrobial susceptibility testing was performed against 14 antimicrobial agents. The presence of virulence genes was studied by PCR. Multilocus sequence typing, *spa* typing and *agr* typing were performed in all *S. aureus* isolates. From the 86 camels tested, 42 staphylococci were isolated, of which there were 11 *S. aureus*, 13 *S. lentus*, 12 *S. sciuri*, 3 *S. xylosus*, *S. epidermidis*, *S. hominis* and *S. chromogenes*. Staphylococci isolates were resistant to penicillin, ciprofloxacin, clindamycin and fusidic acid. All *S. aureus* isolates harbored the *hla*, *hlb* and *hld* virulence genes. *S. aureus* isolates were ascribed to three sequence types (STs) and three *spa* types. All *S. aureus* isolates belonged to *agr* type III. Camels from Gran Canaria used in recreational purposes have a moderate prevalence of *S. aureus* and other coagulase-negative staphylococci. Nevertheless, *S. aureus* isolates are susceptible to almost all antibiotics tested.

## 1. Introduction

Camelids belong to the Camelidae family which comprises the genera *Camelus*, *Lama* and *Vicugna* [1]. The genera *Camelus* includes the species *Camelus dromedarius*, which is the one-humped camel, and the species *Camelus bactrianus*, the two-humped camel [1,2]. *C. dromedarius* is common in Africa, the Middle East, Asia and Australia, while *C. bactrianus* is dispersed in Central Asia, China, East Kazakhstan and Southern Russia [2,3]. In 2020, the camel population was 3,552,527 worldwide, with *C. dromedarius* accounting for approximately 90% of all camels [1,4]. Although Africa contains the largest population of the one-humped dromedary, since 1989, the Canary Islands have been the only region that provides dromedary camels in the European Union [5]. In 2013, the population of camels in the Canary islands was just under 1300 [6]. Camels were mainly used as a source of meat, milk, transportation, agricultural work and racing [7]. However, recently, camel-based tourism has become one of the main attractions in several countries which includes camel riding, trekking, excursions and picture taking [8]. These camel–human close interactive encounters may lead to the transmission of zoonotic agents [5]. Several studies have shown that camels are carriers of many important pathogens such as *Salmonella*, extended-spectrum beta-lactamase-producing *Escherichia coli* and *Pseudomonas aeruginosa*, *Enterococcus* spp. and *Staphylococcus aureus* [4,5,9,10,11].

The genus *Staphylococcus* currently comprises 81 species and subspecies [12]. Both *S. aureus* and coagulase-negative staphylococci, such as *S. epidermidis*, are commensals that colonize the skin and mucosal membranes of humans and several animal species [13]. Studies have shown that camels can also be colonized by *S. aureus*, reporting high carriage rates of around 55% [14,15]. The presence of other species of staphylococci, particularly methicillin-resistant staphylococci (MRS), has not yet been studied much in camels [14]. Furthermore, the prevalence of *S. aureus* and MRS has not yet been studied in camels from Europe. Staphylococci are also opportunistic pathogens that can acquire resistance to several or all classes of antimicrobials, threatening the ability to treat common infections [16]. Methicillin-resistant *S. aureus* (MRSA) is part of the World Health Organization global priority list [17]. Contrary to coagulase-negative staphylococci (CoNS), *S. aureus* produces a wide range of toxins that can act as virulence factors [18]. Nevertheless, lately, there was an emergence of nosocomial infections caused by CoNS which was more often observed in vulnerable patients with an increased risk for infections [19]. Staphylococci have been isolated from a wide range of hosts and environments including humans, livestock, pets, wild animals, air and surface waters [20,21,22,23,24,25]. Animal-associated staphylococci have been reported as infectious agents in humans. These strains pose a zoonotic risk in addition to constituting a reservoir for antimicrobial resistance genes [26]. Therefore, we aimed to investigate the presence of staphylococci in one-humped dromedary camels from the Canary Islands and to characterize the antimicrobial resistance, virulence and genetic lineages of the isolates.

## 2. Materials and Methods

### 2.1. Animals and Bacterial Isolates

**Figure 1:**
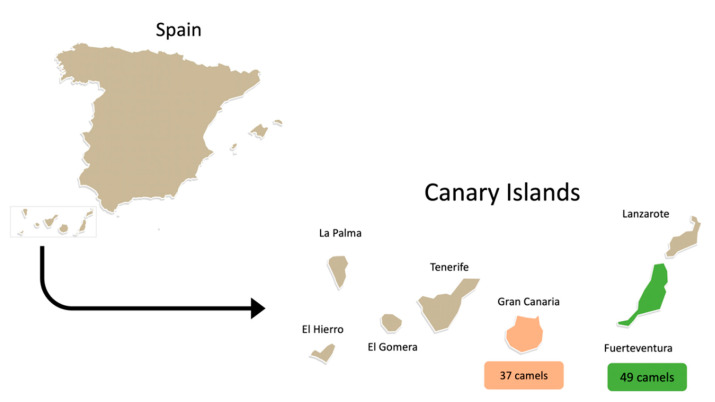
Camel sampling sites: Gran Canaria and Fuerteventura.

Samples were collected from the nostrils and buccal mucosa of 86 one-humped camels from the Canary Islands, making a total of 172 samples as previously described [27]. Samples were collected from 37 camels from Gran Canaria in June 2019 and from 49 camels from Fuerteventura in November 2019 (Figure 1). All camels were domesticated and used in recreational activities. The swabs were placed into tubes containing BHI broth (LiofilChem, Via Scozia, Italy) with 6.5% of NaCl and incubated at 37 °C for 24 h [28]. Then, 150 µL of inoculum was seeded onto Oxacillin Resistance Screening Agar Base Selective Supplement agar (ORSAB; Oxoid, Basingstoke, UK) supplemented with 2 mg/L of oxacillin and Baird-Parker agar (Oxoid, Basingstoke, UK) plates for methicillin-resistant staphylococci (MRS) and *S. aureus* isolation [28]. Up to 4 colonies showing different morphological characteristics were isolated from each plate. Confirmation and identification of staphylococci genera and species were conducted using MALDI-TOF MS [29].

### 2.2. Phenotypic Antimicrobial Resistance

Susceptibility to antimicrobial agents was carried out according to the Kirby–Bauer disk diffusion method against the following 14 antimicrobials (in µg/disk): penicillin G (1 unit), cefoxitin (30), chloramphenicol (30), ciprofloxacin (5), clindamycin (2), erythromycin (15), fusidic acid (10), gentamicin (10), kanamycin (30), linezolid (10), mupirocin (200), tetracycline (30), tobramycin (10) and trimethoprim/sulfamethoxazole (1.25/23.75). The results were analyzed according to the criteria of the European Committee on Antimicrobial Susceptibility Testing (EUCAST) 2018 except for kanamycin that followed the Clinical and Laboratory Standards Institute (CLSI) 2017 guidelines [30,31]. The reference strain *S. aureus* ATCC25923 was used as a quality control strain.

### 2.3. Antimicrobial Resistance and Virulence Genes

DNA extraction was performed as previously described [32]. According to the phenotypic resistance profiles, each isolate was screened for the presence of resistance genes, which included the penicillin resistance gene *bla*Z, the methicillin resistance gene *mec*A, the macrolide and licosamide resistance genes *erm*A, *erm*B, *erm*C, *erm*T, *mph*C, *msr*(A/B), *lnu*A, *lnu*B, *vga*A and *vga*B and the fusidic acid resistance genes *fus*B, *fus*C and *fus*D (Table S2).

All isolates were subjected to PCR for the detection of genes encoding Panton–Valentine leukocidin PVL (*luk*F/*luk*S-PV), hemolysins (*hla*, *hlb* and *hld*), exfoliative toxins (*eta* and *etb*) and toxic shock syndrome toxin (*tst*). Additionally, the scn gene, which is the marker of the immune evasion cluster (IEC) system, was also investigated (Table S2). 

### 2.4. Molecular Typing

The polymorphic X of the *S. aureus* protein A gene (*spa*) was amplified as previously described [33]. The results were analyzed with the Ridom StaphType software (version 1.5, Ridom GmbH, Würzburg, Germany) to determine the *spa* type of each isolate. All *S. aureus* were subjected to multilocus sequence typing (MLST) by amplifying the 7 housekeeping genes (*arc*C, *aro*E, *glp*F, *gmk*, *pta*, *tpi* and *yqi*L) by PCR followed by sequencing as described by Enright et al. [34]. The sequences were submitted to the MLST database (https://pubmlst.org/organisms/staphylococcus-aureus, accessed on 22 November 2021) to obtain the sequence types (STs) and clonal complexes (CCs). *S. aureus* isolates were characterized by *agr* typing (I–IV) by multiplex PCR [35].

## 3. Results and Discussion

The close contact between animals and humans offers favorable conditions for bacterial transmission [36]. The transmission of antimicrobial-resistant staphylococci has been shown between dogs and their owners and livestock and farm workers [37,38]. Therefore, a possible human-to-camel-to-human bacterial transmission may occur during recreational activities. In this study, we analyzed 172 samples recovered from 86 camels from the Canary Islands. A total of 42 staphylococci were isolated from the camels, with 21 staphylococci isolated from nasal samples and the other 21 from oral samples. It has been shown that the animal staphylococcal microbiota varies between anatomical sites due to the different microenvironmental conditions [39,40]. From the 86 camels tested, 11 (12.8%) *S. aureus* were isolated from 10 camels, since 1 camel carried 2 different strains of *S. aureus* (Table 1). *S. aureus* isolates were recovered from six oral samples and five nasal samples. A total of 4 (10.8%) *S. aureus* isolates were recovered from the 37 camels from Gran Canaria and 7 (14.3%) isolates were isolated from the 49 camels from Fuerteventura (Table S1). As far as we know, this is the first study reporting the presence of *S. aureus* and CoNS in camels in Europe. Nevertheless, a few studies have been conducted in healthy camels from the African and Asian continents. The frequency of *S. aureus* isolated from camels in our study is similar to other studies conducted in Egypt and Nigeria and higher than a recent study conducted in Tunisia [4,41,42]. However, two other studies conducted on healthy camels from Saudi Arabia and Algeria reported a much higher frequency of *S. aureus* of 56.2% and 53%, respectively [14,15]. Since most studies conducted on staphylococci from camels showed a prevalence of almost 100% of CoNS colonization, we decided to isolate only methicillin-resistant CoNS (MRCoNS) [14,42]. A total of 31 (18%) MRCoNS were recovered from the 172 samples and identified as *S. lentus* (*n* = 13), *S. sciuri* (*n* = 12), *S. xylosus* (*n* = 3), *S. epidermidis*, *S. chromogenes* and *S. hominis*. From the 100 camels tested in the study by Alzohairy, 8% were positive for MRCoNS, which is a lower frequency than that obtained in our study [14]. Co-carriage of two different species of staphylococci was identified in six animals and co-carriage of three species in two camels. The pattern of co-carriage was as follows: *S. aureus*/*S. sciuri* (*n* = 5), *S. aureus*/*S. chromogenes* (*n* = 1), *S. aureus*/*S. lentus*/*S. sciuri* (*n* = 1) and *S. epidermidis*/*S. hominis*/*S.lentus* (*n* = 1). 

Antimicrobial susceptibility testing was performed in all isolates followed by the screening for antimicrobial resistance and virulence genes. Furthermore, all *S. aureus* isolates were typed by MLST, *spa* typing and *agr* typing. All *S. aureus* isolates were susceptible to all antibiotics tested except for isolate VS3144, which showed resistance to ciprofloxacin, in accordance with the study of Chehida et al. [4]. Other studies conducted in Asia and Africa revealed a higher number of antimicrobial resistances in *S. aureus* from camels [14,15,43]. These differences in results may be due to the different legislation for administering antibiotics to animals established in each continent and country. Furthermore, in our study, none of the *S. aureus* isolates showed methicillin resistance, which contrasts with the high frequency of MRSA found in other studies from Asia and Africa [14,41]. Regarding the presence of virulence genes, all *S. aureus* isolates carried the *hla*, *hlb* and *hld* genes that encode for the alfa-, beta- and delta-hemolysins, which is not surprising since these toxins are present in most *S. aureus* strains, mainly because they are located in very stable regions of chromosomal DNA [44]. Similar results were found in the study of Chehida et al. [4]. However, most studies conducted on camels did not investigate the presence of resistance or virulence genes in staphylococci isolates. *S. aureus* isolates were ascribed to three STs (ST7345, ST88 and ST8) and three *spa* types (t1773, t3221 and t008), showing a low diversity of clonal lineages. Furthermore, *S. aureus* ST7345 and t1773 were isolated from both Gran Canaria and Fuerteventura camels, suggesting either a dominance of these lineages in camels or in the study region. *S. aureus* ST7345 was first described in this study and is a double loci variant of ST130 with mutations in the *aro*E and *pta* loci. *S. aureus* ST130 is frequently associated with ruminants but it has also been isolated from humans and wildlife, usually associated with *mec*C-carrying MRSA isolates [45,46,47]. The *spa* type t1773 was previously reported to be associated with CC130 and common among farm animals and as a frequent cause of ovine mastitis [48,49,50,51]. Three *S. aureus* isolates were ascribed to ST88 which is a relatively rare lineage distributed globally among MRSA and MSSA [52]. This clonal lineage is highly related to community-acquired MRSA strains and is predominant in sub-Saharan Africa [53]. Nevertheless, and in accordance with our results, both *S. aureus* ST130 and ST88 were the predominant clones among samples of healthy camels in Algeria [15]. Furthermore, isolates belonging to ST130 have also been detected in camel’s milk and fermented milk [54,55]. One *S. aureus* isolate was ST8-t008 which is highly related to the CA-MRSA epidemic clone USA300 [21]. Since *S. aureus* ST8-t008 is a classical human pathogen, a possible human-to-animal transmission may have occurred.

Studies reporting the frequency and antimicrobial resistance of *S. aureus* in healthy camels are scarce, but studies showing the frequency and antimicrobial resistance in CoNS are even scarcer. In our study, among the 31 MRCoNS isolates, 13 *S. lentus* and 12 *S. sciuri* were isolated from 12 camels each (Table 2).

In other studies, *S. lentus* has been frequently identified in samples from livestock and from people with occupational exposure to livestock [56,57,58]. *S. sciuri* has a wider host range and is adapted to very different habitats [59,60]. One nasal sample from one camel was positive for *S. lentus* (VS3158) and *S. sciuri* (VS3168), and both isolates showed the same resistance pattern. Another camel simultaneously carried *S. lentus* (VS3155), *S. epidermidis* (VS3152) and *S. chromogenes* (VS3153) in the nasal mucosa. Additionally, the same animal was the only one to carry the same staphylococci species (*S. lentus*) in both the mouth and nose. Nevertheless, the isolates differed in the resistance profile, with the *S. lentus* (VS3166) isolated from the oral sample having resistance to penicillin and clindamycin conferred by the genes *mec*A and *mph*C, and the *S. lentus* isolated from the nasal sample showing only resistance to penicillin. Although *S. epidermidis* and *S. hominis* strains have been isolated from animal samples, these species are the most prevalent CoNS at the clinical level and as part of the normal nasal microbiota of healthy individuals, which may suggest a possible human origin [22,61,62]. All MRCoNS were resistant to penicillin and harbored the *mec*A gene. The presence of the *mec*A gene among staphylococci of the *S. sciuri* group (*S. sciuri*, *S. lentus*, *S. vitulinus* and *S. fleurettii*) is common since it is believed that they played an important role in the origin, evolution and dissemination of *mec*A [63]. None of the MRCoNS showed phenotypic resistance to cefoxitin. In fact, it has been shown that some CoNS carry a homologue of the *mec*A gene which does not confer resistance to β-lactams [64]. Despite all isolates being resistant to penicillin, all isolates lacked the *bla*Z gene, which suggests the presence of other unknown resistance mechanisms or that the breakpoints used for susceptibility testing are not accurate for CoNS [22]. Contrary to what was obtained in the study by Alzohairy, none of our MRCoNS isolates displayed a multidrug resistance profile [14]. Finally, only 7 out of 31 MRCoNS isolates carried virulence genes. The *hld* gene was detected in six isolates, while *hla* was detected in two. *S. epidermidis* was the only isolate that carried both genes. Although CoNS carry fewer virulence genes than *S. aureus* strains, studies have shown that CoNS are a heterogeneous group with distinct virulence potential levels [19,65].

## 4. Conclusions

In this study, a moderate frequency of *S. aureus* and MRCoNS was detected among healthy camels. However, our findings show that, in general, European camels have fewer resistance and virulence genes than healthy camels from Africa and Asia. This study demonstrates a low diversity of *S. aureus.* The predominant lineage was ST7331, followed by ST88, which has already been reported among healthy camels, suggesting that these lineages may be dominant in camels. The carriage of *mec*A-positive staphylococci by camels may be a public health concern since there is a risk of bacterial transmission to humans during recreational activities. Furthermore, since the Canary Islands are the only camel exporter to the EU, camels could constitute a source of zoonotic agents to the rest of the EU.

## Figures and Tables

**Table 1:** Genetic characterization and molecular typing of *S. aureus* isolates from healthy camels.

Isolate	Antimicrobial Resistance		Virulence	Molecular Typing		
	Phenotype	Genotype		ST (CC)	*spa*	*agr*
VS3140	Susceptible	-	*hla, hlb, hld*	7345	t1773	III
VS3141	Susceptible	-	*hla, hlb, hld*	7345	t1773	III
VS3142	Susceptible	-	*hla, hlb, hld*	7345	t1773	III
VS3143	Susceptible	*-*	*hla, hlb, hld*	7345	t1773	III
VS3144	CIP	*-*	*hla, hlb, hld*	7345	t1773	III
VS3145	Susceptible	-	*hla, hlb, hld*	7345	t1773	III
VS3146	Susceptible	-	*hla, hlb, hld*	7345	t1773	III
VS3147	Susceptible	*-*	*hla, hlb, hld*	88	t3221	III
VS3148	Susceptible	-	*hla, hlb, hld*	88	t3221	III
VS3149	Susceptible	-	*hla, hlb, hld*	88	t3221	III
VS3150	Susceptible	-	*hla, hlb, hld*	8 (8)	t008	I

Abbreviations: CIP: ciprofloxacin; ST: sequence type; CC: clonal complex.

**Table 2:** CoNS species identification, antimicrobial resistance and virulence.

	Isolate Species	Antimicrobial Resistance		Virulence Factors
		Phenotype	Genotype	
VS3151	*chromogenes*	PEN	*mec*A	
VS3152	*epidermidis*	PEN	*mec*A	*hla, hld*
VS3153	*hominis*	PEN	*mec*A	
VS3154	*lentus*	PEN, ERY, CD	*mec*A, *mph*C	*hla*
VS3155	*lentus*	PEN	*mec*A	
VS3156	*lentus*	PEN, FD	*mec*A	
VS3157	*lentus*	PEN	*mec*A	
VS3158	*lentus*	PEN	*mec*A	
VS3159	*lentus*	PEN	*mec*A	
VS3160	*lentus*	PEN	*mec*A	
VS3161	*lentus*	PEN	*mec*A	
VS3162	*lentus*	PEN	*mec*A	
VS3163	*lentus*	PEN, FD	*mec*A	*hld*
VS3164	*lentus*	PEN, FD	*mec*A	
VS3165	*lentus*	PEN	*mec*A	
VS3166	*lentus*	PEN, CD	*mec*A, *mph*C	
VS3167	*sciuri*	PEN	*mec*A	
VS3168	*sciuri*	PEN	*mec*A	
VS3169	*sciuri*	PEN	*mec*A	
VS3170	*sciuri*	PEN	*mec*A	
VS3171	*sciuri*	PEN	*mec*A	
VS3172	*sciuri*	PEN	*mec*A	
VS3173	*sciuri*	PEN	*mec*A	*hld*
VS3174	*sciuri*	PEN	*mec*A	
VS3175	*sciuri*	PEN	*mec*A	
VS3176	*sciuri*	PEN	*mec*A	*hld*
VS3177	*sciuri*	PEN	*mec*A	*hld*
VS3178	*sciuri*	PEN, CD, FD	*mec*A, *mph*C	
VS3179	*xylosus*	PEN	*mec*A	
VS3180	*xylosus*	PEN	*mec*A	
VS3181	*xylosus*	PEN	*mec*A	*hld*

Abbreviations: PEN, penicillin; ERY: erythromycin; CD: clindamycin; FD: fusidic acid.

## Data Availability

Not applicable.

## Supplementary Materials

The following supporting information can be downloaded at https://www.mdpi.com/article/10.3390/ani12101255/s1. Table S1: Distribution of staphylococci isolates according to the geographical location and anatomical isolation site; Table S2: Primer pairs used for molecular typing and detection of antimicrobial resistance genes in staphylococci strains. References [35,66,67,68,69,70,71,72,73,74,75,76,77,78,79] are cited in the Supplementary Materials.
