# Supplementary material for: Staphylococcus aureus and Methicillin-Resistant Coagulase-Negative Staphylococci in Nostrils and Buccal Mucosa of Healthy Camels Used for Recreational Purposes
Source: Animals (Basel). 2022 May 13;12(10):1255. doi: 10.3390/ani12101255 (PMC9138023; doi:10.3390/ani12101255)
Supplement: Supplementary file 1 [file animals-12-01255-s001.zip › animals-1677080-supplementary.pdf]

Staphylococcus aureus and Methicillin-Resistant Coagulase-Negative Staphylococci in Nostrils and Buccal Mucosa of Healthy Camels Used for Recreational Purposes

Vanessa Silva, Manuela Caniça, Vera Manageiro, Newton Verbisck, María Teresa Tejedor-Junco, Margarita González-Martin, Juan Alberto Corbera, Patrícia Poeta and Gilberto Igrejas

Table S1. Distribution of staphylococci isolates according to the geographical location and anatomical isolation site.

Animal	Location	Isolation Site	Isolate
Camel 1	Fuerteventura	Nasal	*S. aureus* (VS3145)
		Nasal	*S. chromogenes* (VS3151)
Camel 2	Gran Canaria	Nasal	*S. epidermidis* (VS3152)
		Nasal	*S. hominis* (VS3153)
		Nasal	*S. lentus* (VS3155)
		Oral	*S. lentus* (VS3166)
Camel 3	Gran Canaria	Nasal	*S. lentus* (VS3157)
Camel 4	Fuerteventura	Oral	*S. lentus* (VS3154)
Camel 5	Gran Canaria	Oral	*S. lentus* (VS3156)
Camel 6	Gran Canaria	Oral	*S. aureus* (VS3140)
		Nasal	*S. lentus* (VS3158)
		Nasal	*S. sciuri* (VS3168)
Camel 7	Fuerteventura	Oral	*S. lentus* (VS3159)
Camel 8	Fuerteventura	Oral	*S. lentus* (VS3165)
Camel 9	Fuerteventura	Oral	*S. lentus* (VS3162)
Camel 10	Fuerteventura	Oral	*S. lentus* (VS3160)
Camel 11	Fuerteventura	Oral	*S. lentus* (VS3161)
Camel 12	Fuerteventura	Oral	*S. lentus* (VS3164)
Camel 13	Fuerteventura	Oral	*S. lentus* (VS3163)
Camel 14	Gran Canaria	Oral	*S. sciuri* (VS3167)
Camel 15	Gran Canaria	Oral	*S. sciuri* (VS3170)
Camel 16	Gran Canaria	Oral	*S. aureus* (VS3141)
		Nasal	*S. sciuri* (VS3169)
Camel 17	Fuerteventura	Oral	*S. aureus* (VS3142)
		Nasal	*S. sciuri* (VS3171)
Camel 18	Fuerteventura	Oral	*S. aureus* (VS3146)
		Nasal	*S. sciuri* (VS3172)
Camel 19	Fuerteventura	Oral	*S. sciuri* (VS3173)
Camel 20	Gran Canaria	Nasal	*S. sciuri* (VS3174)
Camel 21	Gran Canaria	Nasal	*S. sciuri* (VS3175)
Camel 22	Fuerteventura	Oral	*S. aureus* (VS3143)
		Nasal	*S. sciuri* (VS3176)
Camel 23	Fuerteventura	Oral	*S. aureus* (VS3144)
		Nasal	*S. sciuri* (VS3177)
Camel 24	Fuerteventura	Oral	*S. sciuri* (VS3178)
Camel 25	Fuerteventura	Nasal	*S. xylosus* (VS3179)
Camel 26	Fuerteventura	Nasal	*S. xylosus* (VS3180)
Camel 27	Fuerteventura	Oral	*S. xylosus* (VS3181)
Camel 28	Fuerteventura	Nasal	*S. aureus* (VS3148)
Camel 29	Gran Canaria	Nasal	*S. aureus* (VS3149)
		Nasal	*S. aureus* (VS3150)
Camel 30	Fuerteventura	Nasal	*S. aureus* (VS3147)

Table S2. Primer pairs used for molecular typing and detection of antimicrobial resistance genes in staphylococci strains.

Gene (amplicon size)	Sequence (5′ › 3′)	Conditions	Reference
*mec*A (527 bp)	F: GGGATCATAGCGTCATTATTC<br>R: AACGATTGTGACACGATAGCC	94 °C 5 min 1 cycle<br>94 °C 30 s<br>55 °C 30 s 30 cycles<br>72 °C 1 min<br>72 °C 10 min 1 cycle	[1]
*bla*Z (772 bp)	F: CAGTTCACATGCCAAAGAG<br>R: TACACTCTTGGCGGTTTC	94 °C 3 min 1 cycle<br>94 °C 1 min<br>50 °C 1 min 30 cycles<br>72 °C 2 min<br>72 °C 5 min 1 cycle	[2]
*erm*A (645 bp)	F: TCTAAAAAGCATGTAAAAGAA<br>R: CTTCGATAGTTTATTAATATTAG		[2]
*erm*B (639 bp)	F: GAAAAGTACTCAACCAAATA<br>R: AGTAACGGTACTTAAATTGTTTA	93 °C 3 min 1 cycle<br>93 °C 1 min<br>52 °C 1 min 35 cycles<br>72 °C 1 min<br>72 °C 5 min 1 cycle	[3]
*erm*C (642 bp)	F: TCAAAACATAATATAGATAAA<br>R: GCTAATATTGTTTAAATCGTCAAT		[4]
*erm*T (200 bp)	F: CCGCCATTGAAATAGATCCT<br>R: TTCTGTAGCTGTGCTTTCAAAAA	94 °C 3 min 1 cycle<br>94 °C 1 min<br>55 °C 1 min 30 cycles<br>72 °C 1 min<br>72 °C 5 min 1 cycle	[5]
msr(A/B) (399 bp)	F: GCAAATGGTGTAGGTAAGACAACT<br>R: ATCATGTGATGTAAACAAAAT	95 °C 3 min 1 cycle<br>93 °C 30 s<br>55 °C 2 min 35 cycles<br>72 °C 1.5 min<br>72 °C 5 min 1 cycle	[6]
mph(C) (900 bp)	F: ATGACTCGACATAATGAAAT<br>R: CTACTCTTTCATACCTAACTC	94 °C 3 min 1 cycle<br>94 °C 1 min<br>45 °C 1 min 30 cycles<br>72 °C 1 min<br>72 °C 5 min 1 cycle	[2]

lnu(A) (323 bp)	F: GGTGGCTGGGGGGTAGATGTATTAACTGG<br>R: GCTTCTTTTGAAATACATGGTATTTTTCGATC	94 °C 2 min 1 cycle<br>94 °C 30 s<br>57 °C 30 s 30 cycles<br>72 °C 1 min<br>72 °C 10 min 1 cycle	[7]
lnu(B) (944 bp)	F: CCTACCTATTGTTTGTGGAA<br>R: ATAACGTTACTCTCCTATTC	94 °C 5 min 1 cycle<br>94 °C 45 s<br>54 °C 45 s 30 cycles<br>72 °C 1 min<br>72 °C 5 min 1 cycle	[8]
vga(A) (1264 bp)	F: AGTGGTGGTGAAGTAACACG<br>R: GGTTCAATACTCAATCGACTGAG	94 °C 3 min 1 cycle<br>94 °C 1 min<br>56 °C 1 min 30 cycles<br>72 °C 1 min<br>72 °C 5 min 1 cycle	[9]
vga(B) (576 pb)	F: TGACAATATGAGTGGTGGTG<br>R: GCGACCATGAAATTGCTCTC	94 °C 1 min 1 cycle<br>94 ºC 1 min<br>55 °C 2 min 30 cycles<br>72 °C 2 min<br>72 °C 10 min 1 cycle	[10]
*fus*B (431 bp)	F: CTATAATGATATTAATGAGATTTTTGG<br>R: TTTTTACATATTGACCATCCGAATTGG	94 °C 3 min 1 cycle<br>94 °C 30 s<br>57 °C 30 s 25 cycles<br>72 °C 45 s<br>72 °C 7 min 1 cycle	[11]
*fus*C (332 bp)	F: TTAAAGAAAAAGATATTGATATCTCGG<br>R: TTTACAGAATCCTTTTACTTTATTTGG		
*fus*D (456 bp)	F: AATTCGGTCAACGATCCC<br>R: GCCATCATTGCCAGTACG		[12]
hla	F: CTGATTACTATCCAAGAAATTCGATTG<br>R: CTTTCCAGCCTACTTTTTTATCAGT		[13]
hlb	F: GTGCACTTACTGACAATAGTGC<br>R: GTTGATGAGTAGCTACCTTCAGT	94 °C 7 min 1 cycle<br>94 °C 30 s<br>57 °C 30 s 30 cycles<br>72 °C 30 s<br>72 °C 10 min 1 cycle	[13]
eta (190 bp)	F: ACTGTAGGAGCTAGTGCATTTGT<br>R: TGGATACTTTTGTCTATCTTTTTCATCAAC		[13]
etb (612 bp)	F: CAGATAAAGAGCTTTATACACACATTAC<br>R: AGTGAACTTATCTTTCTATTGAAAAACACTC		[13]
tst (180 bp)	F: TTCACTATTTGTAAAAGTGTCAGACCCACT<br>R: TACTAATGAATTTTTTTATCGTAAGCCCTT		[13]

lukF/lukS-PV (443 bp)	F: ATCATTAGGTAAAATGTCTGGACATGATCCA<br>R: GCATCAAGTGTATTGGATAGCAAAAGC		[14]
scn (257 bp)	F: AGCACAAGCTTGCCAACATCG<br>R: TTAATATTTACTTTTTAGTGC	95 °C 3 min 1 cycle<br>94 °C 30 s	[15]
chp (366 bp)	F: TTTACTTTTGAACCGTTTCCTAC<br>R: CGTCCTGAATTCTTAGTATGCATATTCATTAG	53 °C 30 s 30 cycles<br>72 °C 2 min	[15]
sak (223 bp)	F: AAGGCGATGACGCGAGTTAT<br>R: GCGCTTGGATCTAATTCAAC	72 °C 6 min 1 cycle	[15]
sea (344 bp)	F: AGATCATTCGTGGTATAACG<br>R: TTAACCGAAGGTTCTGTAGA		[15]
sep (196 bp)	F: AATCATAACCAACCGAATCA<br>R: TCATAATGGAAGTGCTATAA		[15]

References

1. Zhang, K.; Sparling, J.; Chow, B.L.; Elsayed, S.; Hussain, Z.; Church, D.L.; Gregson, D.B.; Louie, T.; Conly, J.M. New quadriplex PCR assay for detection of methicillin and mupirocin resistance and simultaneous discrimination of Staphylococcus aureus from coagulase-negative staphylococci. *J. Clin. Microbiol.* **2004**, *42*, 4947–4955, doi:10.1128/JCM.42.11.4947-4955.2004.
2. Schnellmann, C.; Gerber, V.; Rossano, A.; Jaquier, V.; Panchaud, Y.; Doherr, M.G.; Thomann, A.; Straub, R.; Perreten, V. Presence of New mecA and mph(C) Variants Conferring Antibiotic Resistance in Staphylococcus spp. Isolated from the Skin of Horses before and after Clinic Admission. *J. Clin. Microbiol.* **2006**, *44*, 4444–4454, doi:10.1128/JCM.00868-06.
3. Shopsin, B.; Mathema, B.; Alcabes, P.; Said-Salim, B.; Lina, G.; Matsuka, A.; Martinez, J.; Kreiswirth, B.N. Prevalence of agr specificity groups among Staphylococcus aureus strains colonizing children and their guardians. *J. Clin. Microbiol.* **2003**, *41*, 456–459, doi:10.1128/JCM.41.1.456-459.2003.
4. Sutcliffe, J.; Grebe, T.; Tait-Kamradt, A.; Wondrack, L. Detection of erythromycin-resistant determinants by PCR. *Antimicrob. Agents Chemother.* **1996**, *40*, 2562–2566, doi:10.1128/AAC.40.11.2562.
5. Gómez-Sanz, E.; Torres, C.; Lozano, C.; Fernández-Pérez, R.; Aspiroz, C.; Ruiz-Larrea, F.; Zarazaga, M. Detection, Molecular Characterization, and Clonal Diversity of Methicillin-Resistant Staphylococcus aureus CC398 and CC97 in Spanish Slaughter Pigs of Different Age Groups. *Foodborne Pathog. Dis.* **2010**, *7*, 1269–1277, doi:10.1089/fpd.2010.0610.
6. Wondrack, L.; Massa, M.; Yang, B. V.; Sutcliffe, J. Clinical strain of Staphylococcus aureus inactivates and causes efflux of macrolides. *Antimicrob. Agents Chemother.* **1996**, *40*, 992–998.
7. Lina, G.; Quaglia, A.; Reverdy, M.E.; Leclercq, R.; Vandenesch, F.; Etienne, J. Distribution of genes encoding resistance to macrolides, lincosamides, and streptogramins among staphylococci. *Antimicrob. Agents Chemother.* **1999**, *43*, 1062–1066, doi:10.1128/AAC.43.5.1062.
8. Bozdogan, B.; Berrezouga, L.; Kuo, M.S.; Yurek, D.A.; Farley, K.A.; Stockman, B.J.; Leclercq, R. A new resistance gene, linB, conferring resistance to lincosamides by nucleotidylation in Enterococcus faecium HM1025. *Antimicrob. Agents Chemother.* **1999**, *43*, 925–929.

9. Lozano, C.; Aspiroz, C.; Rezusta, A.; Gómez-Sanz, E.; Simon, C.; Gómez, P.; Ortega, C.; Revillo, M.J.; Zarazaga, M.; Torres, C. Identification of novel vga (A)-carrying plasmids and a Tn5406-like transposon in meticillin-resistant Staphylococcus aureus and Staphylococcus epidermidis of human and animal origin. *Int. J. Antimicrob. Agents* **2012**, *40*, 306–312.
10. Hammerum, A.M.; Jensen, L.B.; Aarestrup, F.M. Detection of the satA gene and transferability of virginiamycin resistance in Enterococcus faecium from food-animals. *FEMS Microbiol. Lett.* **1998**, *168*, 145–151, doi:10.1111/j.1574-6968.1998.tb13267.x.
11. McLaws, F.; Chopra, I.; O'Neill, A.J. High prevalence of resistance to fusidic acid in clinical isolates of Staphylococcus epidermidis. *J. Antimicrob. Chemother.* **2008**, *61*, 1040–1043, doi:10.1093/jac/dkn071.
12. Chen, H.-J.; Hung, W.-C.; Tseng, S.-P.; Tsai, J.-C.; Hsueh, P.-R.; Teng, L.-J. Fusidic acid resistance determinants in Staphylococcus aureus clinical isolates. *Antimicrob. Agents Chemother.* **2010**, *54*, 4985–4991, doi:10.1128/AAC.00523-10.
13. Jarraud, S.; Mougel, C.; Thioulouse, J.; Lina, G.; Meugnier, H.; Forey, F.; Etienne, J.; Vandenesch, F.; Nesme, X. Relationships between Staphylococcus aureus Genetic Background, Virulence Factors, agr Groups (Alleles), and Human Disease. *Infect. Immun.* **2002**, *70*, 631–641, doi:10.1128/IAI.70.2.631.
14. Lina, G.; Piemont, Y.; Godail-Gamot, F.; Bes, M.; Peter, M.-O.; Gauduchon, V.; Vandenesch, F.; Etienne, J. Involvement of Panton-Valentine Leukocidin - Producing Staphylococcus aureus in Primary Skin Infections and Pneumonia. *Clin. Infect. Dis.* **1999**, *29*, 1128–1132, doi:10.1086/313461.
15. van Wamel, W.J.B.; Rooijakkers, S.H.M.; Ruyken, M.; van Kessel, K.P.M.; van Strijp, J.A.G. The innate immune modulators staphylococcal complement inhibitor and chemotaxis inhibitory protein of Staphylococcus aureus are located on beta-hemolysin-converting bacteriophages. *J. Bacteriol.* **2006**, *188*, 1310–1315, doi:10.1128/JB.188.4.1310-1315.2006.
